# Supplementary material for: Physiological temperature drives TRPM4 ligand recognition and gating
Source: Nature. 2024 May 15;630(8016):509–15. doi: 10.1038/s41586-024-07436-7 (PMC11168932; doi:10.1038/s41586-024-07436-7)
Supplement: Supplementary file 2 — Reporting Summary [file 41586_2024_7436_MOESM2_ESM.pdf]

nature portfolio

Corresponding author(s): Juan Du, Wei Lu

Last updated by author(s): Apr 9, 2024

Reporting Summary

Nature Portfolio wishes to improve the reproducibility of the work that we publish. This form provides structure for consistency and transparency in reporting. For further information on Nature Portfolio policies, see our Editorial Policies and the Editorial Policy Checklist.

Statistics

For all statistical analyses, confirm that the following items are present in the figure legend, table legend, main text, or Methods section.

n/a	Confirmed	
☐	☒	The exact sample size (n) for each experimental group/condition, given as a discrete number and unit of measurement
☐	☒	A statement on whether measurements were taken from distinct samples or whether the same sample was measured repeatedly
☐	☒	The statistical test(s) used AND whether they are one- or two-sided<br>*Only common tests should be described solely by name; describe more complex techniques in the Methods section.*
☒	☐	A description of all covariates tested
☐	☒	A description of any assumptions or corrections, such as tests of normality and adjustment for multiple comparisons
☐	☒	A full description of the statistical parameters including central tendency (e.g. means) or other basic estimates (e.g. regression coefficient) AND variation (e.g. standard deviation) or associated estimates of uncertainty (e.g. confidence intervals)
☐	☒	For null hypothesis testing, the test statistic (e.g. F, t, r) with confidence intervals, effect sizes, degrees of freedom and P value noted<br>*Give P values as exact values whenever suitable.*
☒	☐	For Bayesian analysis, information on the choice of priors and Markov chain Monte Carlo settings
☒	☐	For hierarchical and complex designs, identification of the appropriate level for tests and full reporting of outcomes
☒	☐	Estimates of effect sizes (e.g. Cohen's d, Pearson's r), indicating how they were calculated

Our web collection on statistics for biologists contains articles on many of the points above.

Software and code

Policy information about availability of computer code

Data collection	SerialEM 4.15, ClampFit 11.0.3
Data analysis	Relion-4.0, Cryosparc-v2, Motioncor2-1.1.0, Ctffind-4.1.10, Gautomatch-0.56, Topaz-0.2.5, Coot-0.8.9.2, Phenix-1.20.1, UCSF chimeraX-0.91, Hole-2.2005, Pymol-2.3.2, ClamFit_11.0.3, GraphPad Prism 7, OriginPro 2024, eLBOW-1.20.1

For manuscripts utilizing custom algorithms or software that are central to the research but not yet described in published literature, software must be made available to editors and reviewers. We strongly encourage code deposition in a community repository (e.g. GitHub). See the Nature Portfolio guidelines for submitting code & software for further information.

Data

Policy information about availability of data

All manuscripts must include a data availability statement. This statement should provide the following information, where applicable:

- Accession codes, unique identifiers, or web links for publicly available datasets
- A description of any restrictions on data availability
- For clinical datasets or third party data, please ensure that the statement adheres to our policy

Cryo-EM density maps have been deposited in the Electron Microscopy Data Bank (EMDB) under accession numbers EMD-44360 (Ca2+–TRPM4WARM), EMD-44361 (Ca2+–TRPM4WARM Subunit), EMD-44362 (Ca2+/DVT–TRPM4WARM), EMD-44363 (Ca2+/DVT–TRPM4WARM Subunit), EMD-44364 (Ca2+/ATP–TRPM4WARM), EMD-44365 (Ca2+/ATP–TRPM4WARM Subunit), EMD-44366 (Ca2+–TRPM4COLD), EMD-44367 (EDTA–TRPM4), EMD-44368 (Ca2+TRPM4(E396A))

and EMD-44369 (Ca2+/ATP–TRPM4WARM + DVT). Structure models have been deposited in the RCSB Protein Data Bank under accession codes 9B8W (Ca2+–TRPM4WARM), 9B8X (Ca2+–TRPM4WARM Subunit), 9B8Y (Ca2+/DVT–TRPM4WARM), 9B8Z (Ca2+/DVT–TRPM4WARM Subunit), 9B90 (Ca2+/ATP–TRPM4WARM), 9B91 (Ca2+/ATP–TRPM4WARM Subunit), 9B92 (Ca2+–TRPM4COLD), 9B93 (EDTA–TRPM4) and 9B94 (Ca2+TRPM4(E396A)).

Research involving human participants, their data, or biological material

Policy information about studies with human participants or human data. See also policy information about sex, gender (identity/presentation), and sexual orientation and race, ethnicity and racism.

Reporting on sex and gender	Does not apply
Reporting on race, ethnicity, or other socially relevant groupings	Does not apply
Population characteristics	Does not apply
Recruitment	Does not apply
Ethics oversight	Does not apply

Note that full information on the approval of the study protocol must also be provided in the manuscript.

Field-specific reporting

Please select the one below that is the best fit for your research. If you are not sure, read the appropriate sections before making your selection.

☒ Life sciences ☐ Behavioural & social sciences ☐ Ecological, evolutionary & environmental sciences

For a reference copy of the document with all sections, see nature.com/documents/nr-reporting-summary-flat.pdf

Life sciences study design

All studies must disclose on these points even when the disclosure is negative.

Sample size	The sample sizes of the cryo-EM data were not predetermined, but were determined/limited by the available time of the microscope. These sample sizes were large enough as they allowed for the reconstruction of cryo-EM maps of sufficiently high quality to draw the conclusions of this study.
Data exclusions	During cryo-EM data processing, particles that clearly did not represent the target protein or did not show high-resolution features were discarded. The data processing workflows are summarized in the Extended Data Figures. These workflows were predefined and represent common practices in cryo-EM image processing.
Replication	For the cryo-EM studies, initial data collection was performed on a 200 kV Arctica microscope. Subsequently, in order to validate the structural findings and to improve the quality of the data, larger datasets were collected on a 300 kV Titan Krios microscope. All attempts to replicate the structural findings were successful. For patch-clamp experiment, independent measurements/replicates are indicated in the figure legend.
Randomization	This study did not allocate experimental groups, therefore, no randomization was necessary.
Blinding	Blinding was not applicable to single-particle cryo-EM studies.

Reporting for specific materials, systems and methods

We require information from authors about some types of materials, experimental systems and methods used in many studies. Here, indicate whether each material, system or method listed is relevant to your study. If you are not sure if a list item applies to your research, read the appropriate section before selecting a response.

Materials & experimental systems

n/a	Involved in the study	
☒	☐	Antibodies
☐	☒	Eukaryotic cell lines
☒	☐	Palaeontology and archaeology
☒	☐	Animals and other organisms
☒	☐	Clinical data
☒	☐	Dual use research of concern
☒	☐	Plants

Methods

n/a	Involved in the study	
☒	☐	ChIP-seq
☒	☐	Flow cytometry
☒	☐	MRI-based neuroimaging

Eukaryotic cell lines

Policy information about cell lines and Sex and Gender in Research

Cell line source(s)	tsa201 cells from Sigma-Aldrich
Authentication	Cells are obtained from the vendor and is not independently authenticated
Mycoplasma contamination	Cells are tested free from Mycoplasma contamination
Commonly misidentified lines (See ICLAC register)	None

Plants

Seed stocks	Does not apply
Novel plant genotypes	Does not apply
Authentication	Does not apply
